# Comparative study of the morphological characteristics of *Phoenix dactylifera* L. cultivars in Al-Madinah Al-Munawarah-Saudi Arabia

**DOI:** 10.1186/s12870-022-03841-0

**Published:** 2022-09-26

**Authors:** Meaad F. Alaida, Amal Y. Aldhebiani

**Affiliations:** 1 Department of Biological Sciences, Faculty of Sciences, King Abdulaziz University, Jeddah, Saudi Arabia; 2 Biological Sciences Department , Faculty of Science , Aljouf University, Aljouf-Skaka, Saudi Arabia

**Keywords:** *Phoenix dactylifera* cultivars, Date palm, Al-Madinah Al-Munawarah, Morphological characters, MVSP, UPGMA, Saudi Arabia

## Abstract

**Background:**

*Phoenix dactylifera* L. belongs to the subfamily Coryphoideae. Saudi Arabia is the third producing country of dates in the world with over a million tons of dates every year. *P. dactylifera* is one of the most important species that grows in Al-Madinah and has cultivars that are distinguished by their appearance and taste.

**Results:**

This study aimed to investigate the importance of morphology among *P. dactylifera* cultivars by using statistical analysis and the ability to identify the cultivars just by looking at them in the obvious characters of palms. Plant specimens were collected from different areas in the Al-Madinah region. All the data obtained from morphology were transferred to numerical characters and used in the multivariate statistical package (MVSP) to study the similarity between the cultivars and give phenetic clusters. One-way ANOVA test and the least significant difference test (LSD) were used to find the significant differences among cultivars in *p* = 0.05. The numerical data that was recorded indicated significant differences among cultivars. Principal coordinates analysis and cluster analysis (UPGMA) were utilized to study the distance of similarities and differences between cultivars.

**Conclusion:**

The most distinguishing characteristics were fruit and seed, and the least characteristic was the trunk. However, the features of spine, frond and leaflet were also important in distinguishing between cultivars.

## Background

*Phoenix dactylifera* belongs to the subfamily Coryphoideae and the tribe of Phoeniceae. Palms are found on coasts in tropical and sub-tropical ecological zones, the Arabian deserts and Africa [1]. Dates are a major source of revenue and a portion of basic food for local inhabitants in regions where they are planted. As a result, they have played important roles in those regions’ economies, societies and environments, for example, the planting of dates has a significant impact on Middle Eastern history, and there has been no way for the local population to survive in the desert without palm fruits [2]. The two primary production locations for dates are the Middle East and North Africa [3]. In addition, the leaves are used in making mats, baskets and other furniture. Moreover, palm trees are used as ornamental plants. Date palms are an essential component of biodiversity in hostile desert stings because they are a crucial special that has adapted to the extreme climatic conditions of arid zones [4].

Palm trees are characterized by either solitary, clustered or dioecious; leaves are pinnate with armed petiole by narrow spines, modified leaflets; the blade is split into multiple single folded induplicate leaflets; the inflorescences are arranged in a single order; flowers borne solitary in a spiral along the axis by a tiny bract; male flowers with fused tepals in two whorls while female flowers have three outer tepals fused into a cup with three inner tepals free and imbricate; stamens are six set on inner tepals; carpels are free three with short flashy stigmas, and they mostly developing only one carpel per fruit; have has smooth epicarp, flashy mesocarp and thin endocarp and seeds have a longitudinal groove running its length [5].

The regions in Arab Gulf and North Africa countries are characterized by specific date palm cultivars. Saudi Arabia is the third producing country of dates in the world with over a million tons of dates every year [6]. There are around 400 cultivars in Saudi Arabia [7]. The date palm cultivars found in Saudi Arabia are unique. In addition, each region is distinguished by distinctive cultivars. For example, the famous cultivars are Khalas in the Eastern region, Sukari in Qassim, Hilwah Aljouf in Aljouf, and Ajwah in Al-Madinah Al-Munawarah.

Many studies have examined the morphological characteristics of *P. dactylifera.* The morphological characteristics of 21cultivars in Egypt were characterized, and it was explained the features of trunk, crown, leaves, fruits and seeds in detail to investigate the taxonomic relationship among cultivars [8]. The 14 cultivars from Al-Qassim region-Saudi Arabia we analyzed, and it was compared among these cultivars’ fruits based on shape, variations in color during the three phases of fruit ripening (beser, rutab and tamer), fruits apex and base, as well as the diameter of the fruit cap [4]. The morphological characteristics of some date palm cultivars growing the in Eastern region, Western region and Central region of Saudi Arabia were described based on vegetative and reproductive characteristics [9]. The 20 Emirati dates in the tamar stage were explained and focused on the size, shape, color and texture of fruits, and statistical analyses were performed on the given data to determine similarities and differences among cultivars [10]. The 12 male date palm cultivars in Iraq were studied based on vegetative and floral characteristics, as well as pollen grain vitality and germination percentage, and it was used in cluster analysis for determining the relationship among them [11].

Al-Madinah Al-Munawarah is an important region in the west of Saudi Arabia between longitudes 36°39′ east and latitudes 28°24′ north. It is characterized by a diversity of plants. It is famous for its production of the rose plants, mint varieties and date palm cultivars. As a result, *Phoenix dactylifera* is one of the most significant species found in Al- Madinah with a variety of cultivars that are distinguished by their appearance and taste. Some these cultivars are Ajwah Al-Madinah, Safawi, Barni Al-Madinah, Hilwah Al-Ula, Rothanah Al-Madinah, Segaae, Mabroom, Majdool, Beid, Anbarah and Shalabi. However, cultivars in Al-Madinah region such as Beid, Loun and Hilwah Al-Ula have not been studied previously for their morphological characteristics. Consequently, this study aims to investigate the importance of morphology among *Phoenix dactylifera* cultivars in Al-Madinah Al- Munawarah region by using statistical analysis. In addition, the ability to identify the cultivars just by looking at them in the obvious characters of palms.

## Results

In ANOVA test, the numerical data that was recorded for the date palm cultivars in Al-Madinah Al-Munawarah indicated significant differences among them. Thus, the least significant difference test was used to find the groups that have a significant difference between them in *p* = 0.05.

In MVSP, principal coordinates and cluster analysis (UPGMA) were used to study the distances of similarities and differences between the cultivars.

The differences in traits of cultivars were explained as follows:

### Trunk

The characters of trunks were recorded in Table 1. The largest cultivar was Barni Al-Eis while the smallest was Shalabi. Hilwah Al-Ula and Ajwah were more similar in trunk characters.

**Table 1:** The trunk characters of *Phoenix dactylifera* cultivars

Cultivars	Trunk circumference (cm)	Diameter of Trunk (cm)
Ajwah	218	69.43
Safawi	292	92.99
Shalabi	162	51.59
Rothanah	193	61.46
Barni Al-Madinah	280	89.17
Segaae	310	98.73
Majdool	180	57.32
Loun	228	72.61
Beid	245	78.03
Barni Al-Eis	340	108.28
Anbarah	210	66.88
Hilwah Al-Ula	219	69.75
Altaibat	263	83.76
Mabroom Al-Ula	239	76.11

**Fig. 1:**
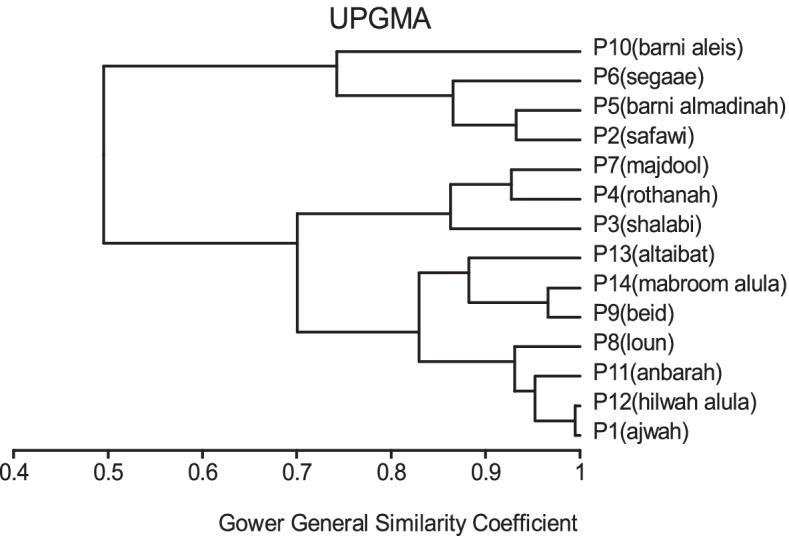
The degree of similarity and difference in the trunk characteristics among *Phoenix dactylifera* cultivars by using cluster analysis

**Fig. 2:**
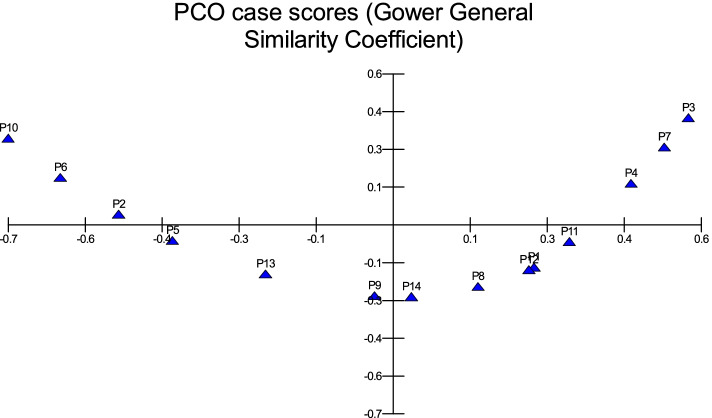
The degree of similarity and difference in the trunk characteristics among *Phoenix dactylifera* cultivars by PCO analysis

In MVSP, the results show that cultivars were classified into two groups in degree (0.496). The first group was divided into three clades: 1- Barni Al-Eis is similar by (0.742), 2- Segaae was similar by (0.865), 3- Barni Al-Madinah and Safawi were more similar by (0.933). The second group was classified into two clades and were similar by (0.700): 1- (a) Majdool and Rothanah were similar by (0.926), (b) Shalabi was similar by (0.862); 2- this subgroup had two clades and were similar by (0.830): (aa) Altaibat was similar in (0.882), (ab) Mabroom Al-Ula and Brid were more related in (0.966), (ba) Loun was similar by (0.931), (bb) Anbarah was similar by (0.952), 3- Hilwah Al-Ula and Ajwah was most closely related in degree (0.994) (Figs. 1 and 2).

### Fronds

**Fig. 3:**
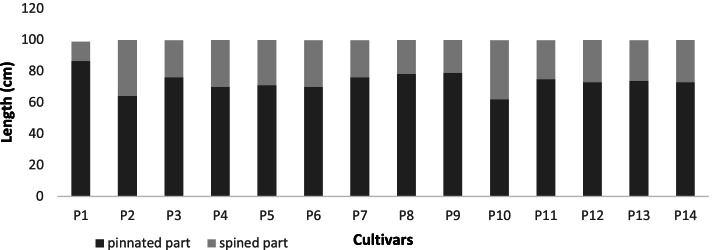
The variation between pinnated and spined parts in *Phoenix dactylifera* cultivars

The characteristics of fronds were recorded in Table 2. Based on the ratio of frond length/width, the broadest frond was Barni Al-Eis (3.5) and the narrowest one was Loun (6.75). the range of frond length was from 545.33 cm (Barni Al-Madinah) to 297 cm (Majdool) while the range of frond width was from 106.17 cm (Barni Al-Madinah) to 55.67 cm (Mabroom Al-Ula). There was a difference among cultivars in the percentage of pinnated part and spined part in the total frond length (Fig. 3). The measure was replicated in six fronds in each palm.

**Table 2:** The frond characters of *Phoenix dactylifera* cultivars

cultivars	Length of Frond (cm) LSD 13.83	Width of Frond (cm) LSD 6.39	The ratio of Frond L/W	Pinnated Part Length (cm) LSD 12.03	Spined Part Length (cm) LSD 3.83	Percentage of pinnated part in total frond length %	Percentage of spined part in total frond length %
Ajwah	361.83 ± 15.01**d**	101.17 ± 5.2**fe**	3.58	312.67 ± 22.92 **g**	44.67 ± 5.01**a**	86.41	12.35
Safawi	421.5 ± 9.27**f**	94.83 ± 6.82**e**	4.44	270.33 ± 6.95**de**	150.5 ± 2.88**i**	64.14	35.71
Shalabi	334.33 ± 15.34**bc**	72.5 ± 3.73**b**	4.61	254.17 ± 12.19**c**	79.33 ± 3.5**c**	76.02	23.73
Rothanah	399.83 ± 11.18**e**	84.33 ± 4.97**d**	4.74	279.67 ± 7.79**e**	119.5 ± 3.78 **g**	69.95	29.89
Barni Al-Madinah	545.33 ± 14.68 **h**	106.17 ± 6.9 **g**	5.14	387.17 ± 10.87**i**	157.5 ± 4.23**j**	71	28.88
Segaae	347 ± 12**c**	79.33 ± 5.5 **cd**	4.37	242.67 ± 8.55**c**	103.5 ± 3.62**e**	69.93	29.83
Majdool	297 ± 9.57**a**	68.17 ± 5.49**b**	4.35	225.5 ± 6.98**b**	70.67 ± 2.58**b**	75.93	23.79
Loun	405 ± 11.31**e**	60 ± 4.43**a**	6.75	316.17 ± 8.91 **g**	88.5 ± 2.43**d**	78.07	21.85
Beid	338.5 ± 17.01**c**	84 ± 6.51**d**	4.03	267 ± 13.52**d**	70.88 ± 3.43**b**	78.88	20.94
Barni Al-Eis	340.17 ± 9.15**c**	97.17 ± 9.24**ef**	3.5	210.67 ± 6.28**a**	128.67 ± 3.14 **h**	61.93	37.83
Anbarah	323.17 ± 7.76**b**	74.17 ± 3.71**bc**	4.36	242.33 ± 5.79**c**	80 ± 2.1**c**	74.99	24.75
Hilwah Al-Ula	406.67 ± 12.11**e**	83 ± 4.05**d**	4.9	296.67 ± 9.05**f**	109.33 ± 3.33**f**	72.95	26.88
Altaibat	472.33 ± 10.61 **g**	102.33 ± 3.9**f**	4.62	348.67 ± 7.45 **h**	122.33 ± 2.8 **g**	73.82	25.9
Mabroom Al-Ula	297.17 ± 8.66**a**	55.67 ± 4.08**a**	5.34	216.83 ± 5.74**ab**	79.83 ± 2.48**c**	72.97	26.86

In ANOVA test, there was a significant difference in all four characters of fronds with a significance of (*p* < 0.001). Least significant difference (LSD) test was calculated to find the groups that have a significant difference among cultivars. The means with the different letters in the same characters were significantly different in *p* = 0.05 (Table 4). The frond character that distinguished among cultivars was spined part length, and it classified the cultivars into ten separate groups (Ajwah), (Majdool, Beid), (Shalabi, Anbarah, Mabroom Al-Ula), (Loun), (Segaae), (Hilwah Al-Ula), (Rothanah, Altaibat), (Barni Al-Eis), (Safawi) and (Barni Al-Madinah). The longest frond of cultivars was Barni Al-Eis while the shortest one was Ajwah.

**Fig. 4:**
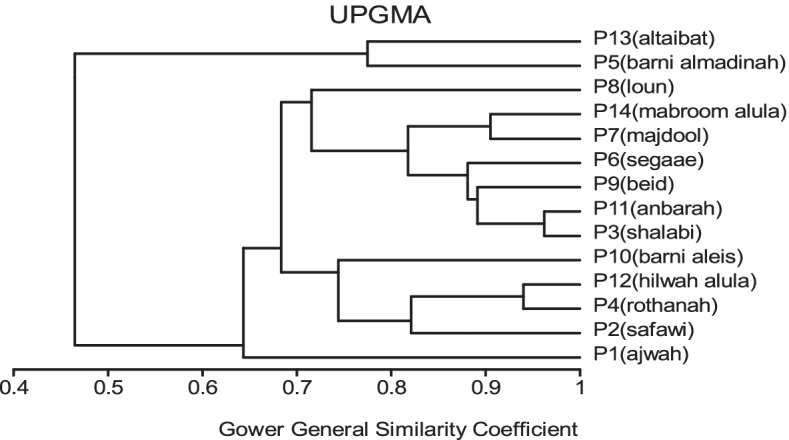
The degree of similarity and difference in the frond characteristics among *Phoenix dactylifera* cultivars by using cluster analysis

**Fig. 5:**
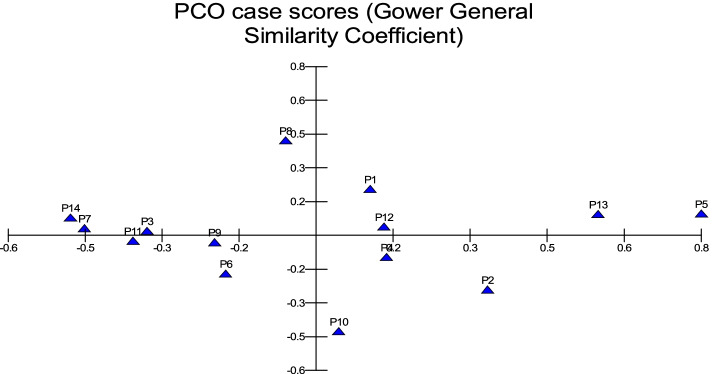
The degree of similarity and difference in the frond characteristics among *Phoenix dactylifera* cultivars by PCO analysis

In MVSP, the results show that cultivars were classified into two groups in degree (0.464). The first group consists of one clade which was Altaibat and Barni Al-Madinah, and they were similar by (0.775). The second group was divided into two clades: 1- this subgroup had two clades and were similar by (0.684): (aa) Loun was similar by (0.715), (ab) also this subgroup was divided into two clades in degree (0.818): (ab1) Mabroom Al-Ula and Majdool were similar by (0.905), (ab2) Segaae was similar by (0.881), Beid was similar by (0.891), Anbarah and Shalabi were similar by (0.962); (ba) this group was divided into two clades:(ba1) Barni Al-Eis was similar by (0.743), (ba2) Hilwah Al-Ula and Rothanah were similar by (0.940), and Safawi was similar by (0.821), 2- Ajwah was similar by (0.664) (Figs. 4 and 5).

### Leaflets

The characteristics of the leaflet were recorded in Table 3. The range of leaflet length was from 62.33 cm (Ajwah) to 34.5 cm (Mabroom Al-Ula) while the range of leaflet width was from 4.9 cm (Safawi) to 2.87 cm (Mabroom Al-Ula). The broadest leaflet was 10.21 (Loun) and the narrowest one was 16.64 (Ajwah) based on the ratio of length /width of leaflets. The colors of leaflet were either light green, dark green or ashy green. In addition, the number of leaflets per frond is different between cultivars. The more density was 216 leaflets (Rothanah) while the less density was 136 leaflets (Mabroom Al-Ula). The measure was replicated in six leaflets in each palm from the middle of the fronds.

**Table 3:** The leaflet characters of *Phoenix dactylifera* cultivars

Cultivars	Color of Leaflets	length of leaflet (cm) LSD 4.25	width of leaflet (cm) LSD 0.43	The ratio of leaflet L/W	Number of leaflets per frond LSD 10.66
Ajwah	Light Green	62.33 ± 3.5**f**	3.78 ± 0.28**bc**	16.49	182 ± 10**de**
Safawi	Light Green	57.67 ± 2.25**f**	4.9 ± 0.2**f**	11.77	183 ± 7**de**
Shalabi	Light Green	52.67 ± 2.66**de**	3.37 ± 0.23**b**	15.63	172 ± 13 **cd**
Rothanah	Ashy Green	54.83 ± 1.72**ef**	4.08 ± 0.53 **cd**	13.44	216 ± 12**f**
Barni Al-Madinah	Ashy Green	54.83 ± 3.49**ef**	4.72 ± 0.21**ef**	11.62	183 ± 7**de**
Segaae	Light Green	50.5 ± 4.23 **cd**	3.45 ± 0.52**b**	14.64	173 ± 11 **cd**
Majdool	Light Green	53 ± 5.02**de**	4.38 ± 0.32**de**	12.1	165 ± 5**c**
Loun	Ashy Green	48 ± 5.83**bc**	4.7 ± 0.21**ef**	10.21	212 ± 10**f**
Beid	Dark Green	47.33 ± 4.08**bc**	3.77 ± 0.56**bc**	12.55	206 ± 13**f**
Barni Al-Eis	Dark Green	58.5 ± 5.54**f**	3.62 ± 0.33**b**	16.16	149 ± 7**b**
Anbarah	Ashy Green	52.83 ± 1.17**de**	3.42 ± 0.15**b**	15.45	148 ± 7**b**
Hilwah Al-Ula	Ashy Green	45.83 ± 2.64**b**	3.63 ± 0.31**b**	12.63	167 ± 9**c**
Altaibat	Dark Green	55.83 ± 4.17**ef**	3.48 ± 0.12**b**	16.04	188 ± 7**e**
Mabroom Al-Ula	Ashy Green	34.5 ± 1.05**a**	2.87 ± 0.7**a**	12.02	136 ± 6**a**

In ANOVA test, there was a significant difference among all three characters of leaflet with a significance of (*p* < 0.001). LSD test was calculated to find the groups that have a significant difference among cultivars. The means of characters with the different letters were significantly different with *p* = 0.05 (Table 3). Leaflet characters classified the cultivars into six separate groups. However, the distinguishing cultivar between them was Mabroom Al-Ula.

**Fig. 6:**
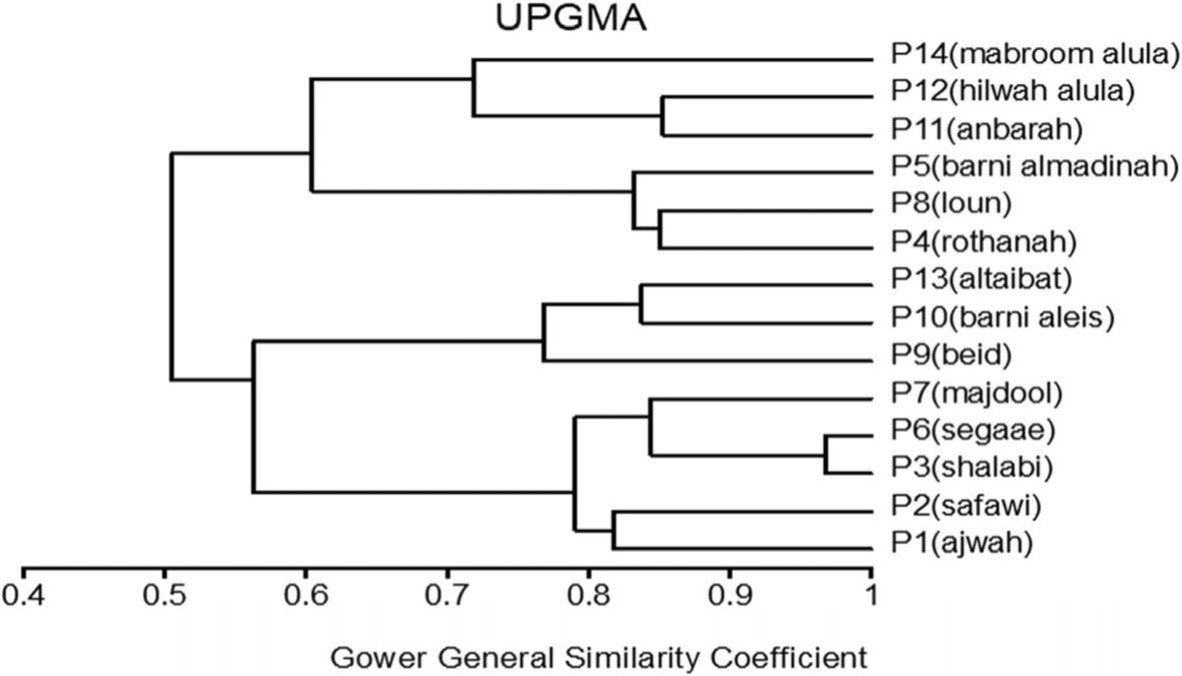
The degree of similarity and difference in the leaflet characteristics among *Phoenix dactylifera* cultivars by using cluster analysis

**Fig. 7:**
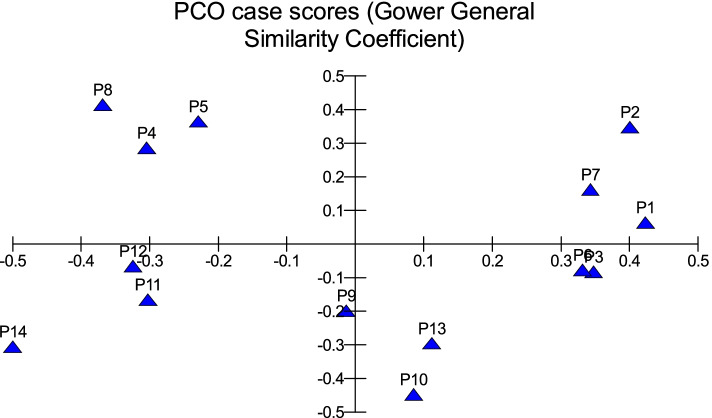
The degree of similarity and difference in the leaflet characteristics among *Phoenix dactylifera* cultivars by PCO analysis

In MVSP, the results show that cultivars were classified into two groups in degree (0.505). The first group was divided into two clades in degree (0.603): 1- Hilwah Al-Ula and Anbarah were similar by (0.852), and Mabroom Alula was similar by (0.719); 2- Loun and Rothanah were similar by (0.850) while Barni Al-Madinah was similar by (0.832). The second group was classified into two clades in degree (0.563): 1- Altaibat and Barni Al-Eis were similar by (0.837) while Beid was similar by (0.767); 2- this subgroup had two clades in degree (0.790), which were: (a) Safawi and Ajwah were similar by (0.817), (b) Segaae and Shalabi were more similar by (0.968) while Majdool was similar by (0.844) (Figs. 6 and 7).

### Spines

The characteristics of spines were recorded in Table 4. The range of spines length was from 18.67 cm (Ajwah) to 10 cm (Barni Al-Eis). The number of spines per frond was different between cultivars. The more density was 19 spines (Barni Al-Madinah) while the less density was ten spines (Anbarah). The measure was replicated in six spines in each palm from the bottom of the frond.

**Table 4:** The spines characters of *Phoenix dactylifera* cultivars

Cultivars	Length of Spines LSD 4.61	Number of Spines per Frond LSD 2.65
Ajwah	18.67 ± 1.53**d**	15 ± 2**def**
Safawi	17.33 ± 4.68 **cd**	17 ± 2**fgh**
Shalabi	11.67 ± 5.05**a**	13 ± 2**bcd**
Rothanah	12.5 ± 3.56**ab**	17 ± 2**fgh**
Barni Al-Madinah	10.5 ± 2.35**a**	19 ± 4 **h**
Segaae	13.17 ± 4.36**abc**	16 ± 2**efg**
Majdool	14.33 ± 4.37**abcd**	18 ± 2**gh**
Loun	18.5 ± 7.12**d**	11 ± 2**ab**
Beid	11.17 ± 3.82**a**	18 ± 2**gh**
Barni Al-Eis	10 ± 1.79**a**	12 ± 2**abc**
Anbarah	11 ± 2.53**a**	10 ± 1**a**
Hilwah Al-Ula	16.5 ± 3.73**bcd**	17 ± 3**fgh**
Altaibat	18.17 ± 2.4**d**	18 ± 2**gh**
Mabroom Al-Ula	16.67 ± 4.89**bcd**	14 ± 3**cde**

In ANOVA test, there was a significant difference among all two characters of spines with a significance of (*p* < 0.001). LSD test was calculated to find the groups that have a significant difference among cultivars. The means of characters with the different letters were significantly different with *p* = 0.05 (Table 6). The number of spines per frond classified the cultivars into eight separate groups while the length of spines classified them into four groups. However, cultivars were no significant differences between others because one cultivar shared with others in groups. The distinguishing cultivar between them was Anbarah.

**Fig. 8:**
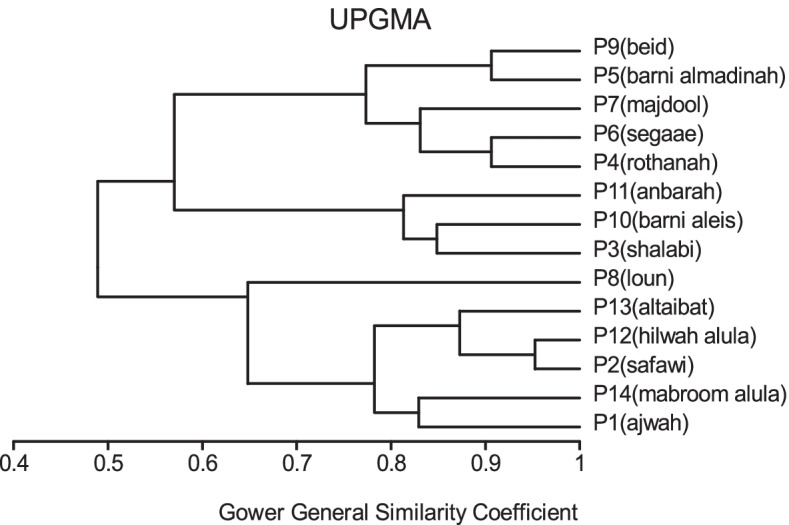
The degree of similarity and difference in the spine characteristics among *Phoenix dactylifera* cultivars by using cluster analysis

**Fig. 9:**
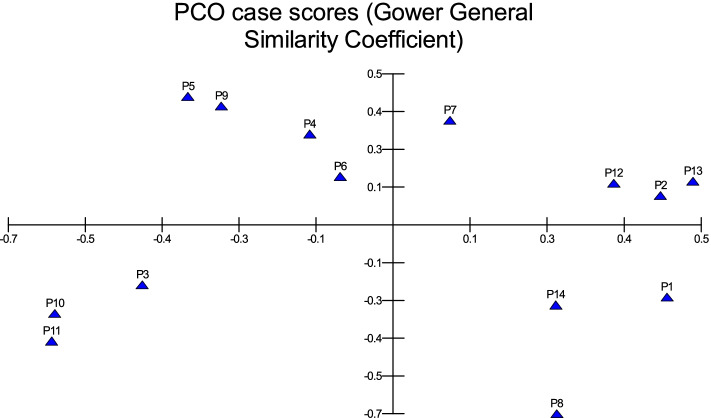
The degree of similarity and difference in the spine characteristics among *Phoenix dactylifera* cultivars by PCO analysis

In MVSP, the results show that cultivars were classified into two groups in degree (0.489). The first group was divided into two clades in degree (0.570):1- this clade was classified into two clades in degree (0.782): (a) Beid and Barni Al-Madinah were similar by (0.906), (b) Segaae and Rothanah were similar by (0.906) while Majdool was similar by (0.830); 2- Barni Al-Eis and Shalabi were similar by (0.848) while Anbarah was similar by (0.813). The second group was classified into two clades in degree: 1- Loun was similar by (0.648); 2- this subgroup had two clades in degree (0.773) which were: (a) Hilwah Al-Ula and Safawi were more similar in (0.952) while Altaibat was similar by (0.872), (b) Mabroom Al-Ula and Ajwah were similar by (0.829) (Figs. 8 and 9).

### Fruits

The characteristics of the fruit were recorded in Table 5. The fruit characters vary between cultivars. The colors of fresh fruit were either red or yellow while black, brown, light brown or reddish brown in dry fruit. The range of fruit length was from 5.3 cm (Anbarah) to 2.78 cm (Beid) while the range of leaflet width was from 2.28 cm (Majdool) to 1.73 cm (Mabroom Al-Ula). In addition, the shapes of the fruit were either globose, oblong, ovoid or linear oblong. The tip of fruit was cordate, shallowly cordate or truncate while the base was rounded or obtuse. Also, the surface was either smooth or rugose. The measure was replicated in ten fruits from each palm.

**Table 5:** The fruit characters of *Phoenix dactylifera* cultivars

Cultivars	Color of Dry Fruit	Color of Fresh Fruit	Surface of Fruit	Base of the Fruit	Tip of the Fruit	Shape	The ratio of Fruit L/W	Fruit Width (cm) LSD 0.22	Fruit Length (cm) LSD 0.31
Ajwah	Black	Red	2.93 ± 0.22**ab**	2.23 ± 0.19**de**	1.31	Globose	Cordate	Rounded	Rugose
Safawi	Reddish Brown	Red	4.05 ± 0.26**d**	2.07 ± 0.29**cde**	1.96	Oblong	Shallowly cordate	Obtuse	Rugose
Shalabi	Brown	Yellow	4.1 ± 0.25**de**	2.08 ± 0.37**cde**	1.97	Oblong	Shallowly cordate	Obtuse	Rugose
Rothanah	Brown	Yellow	2.88 ± 0.16**a**	1.98 ± 0.1**bc**	1.45	Globose	Shallowly cordate	Rounded	Smooth
Barni Al-Madinah	Brown	Yellow	3.95 ± 0.08**d**	2.07 ± 0.16**cde**	1.91	Oblong	Cordate	Obtuse	Smooth
Segaae	Light Brown	Yellow	3.45 ± 0.36**c**	1.85 ± 0.12**ab**	1.86	Ovoid	Cordate	Obtuse	Rugose
Majdool	Reddish Brown	Red	4.82 ± 0.49 **g**	2.28 ± 0.28**e**	2.11	Oblong	Shallowly cordate	Obtuse	Rugose
Loun	Brown	Yellow	3.28 ± 0.18c	1.85 ± 0.1**ab**	1.77	Ovoid	Shallowly cordate	Rounded	Smooth
Beid	Brown	Yellow	2.78 ± 0.15**a**	1.9 ± 0.09**abc**	1.46	Globose	Shallowly cordate	Rounded	Smooth
Barni Al-Eis	Light Brown	Yellow	4.38 ± 0.16**ef**	2.03 ± 0.15**bcd**	2.16	Oblong	Cordate	Obtuse	Rugose
Anbarah	Brown	Red	5.3 ± 0.24 **h**	1.83 ± 0.15**ab**	2.9	Linear-Oblong	Truncate	Obtuse	Rugose
Hilwah Al-Ula	Reddish Brown	Red	4.17 ± 0.27**def**	2.2 ± 0.09**de**	1.9	Oblong	Truncate	Rounded	Rugose
Altaibat	Light Brown	Yellow	3.22 ± 0.28**bc**	1.73 ± 0.23**a**	1.86	Ovoid	Shallowly cordate	Obtuse	Rugose
Mabroom Al-Ula	Reddish Brown	Red	4.4 ± 0.33**f**	1.73 ± 0.15**a**	2.54	Linear-Oblong	Truncate	Obtuse	Rugose

In ANOVA test, there was a significant difference among all two characters of fruit with a significance of (*p* < 0.001). LSD test was calculated to find the groups that have a significant difference among cultivars. The means of characters with the different letters were significantly different with *p* = 0.05 (Table 5). The fruit length was classified the cultivars into eight different groups (Ajwah, Rothanah, Beid), (Ajwah, Altaibat), (Segaae, Loun, Altaibat), (Safawi, Barni Al-Madinah, Hilwah Al-Ula), (Shalabi, Barni Al-Eis, Hilwah Al-Ula), (Barni Al-Eis, Hilwah Al-Ula, Mabroom Al-Ula), (Majdool), (Anbarah) while the width of fruit had divided them into five groups. However, cultivars were no significant differences between others in fruit width because one cultivar shared with others in groups. The distinguishing cultivars were Majdool and Anbarah.

**Fig. 10:**
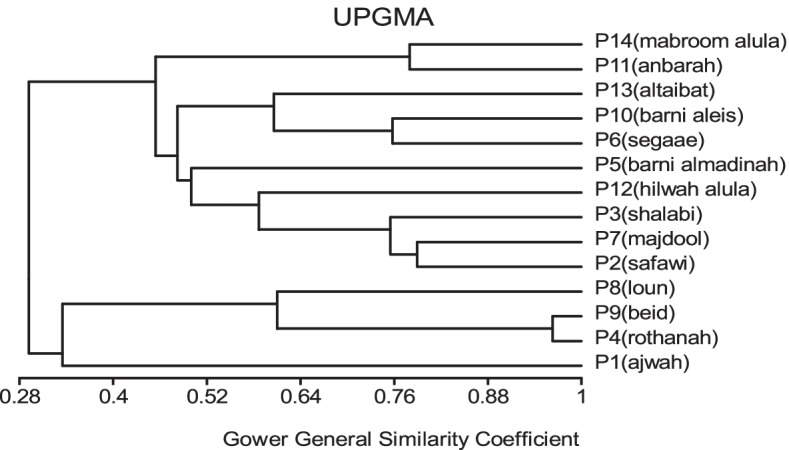
The degree of similarity and difference in the fruit characteristics among *Phoenix dactylifera* cultivars by using cluster analysis

**Fig. 11:**
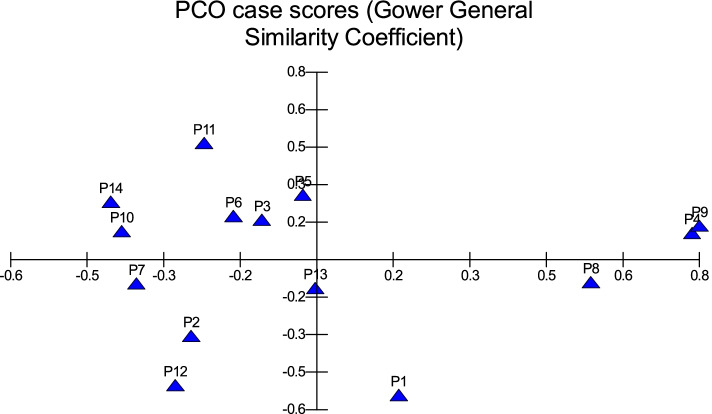
The degree of similarity and difference in the fruit characteristics among *Phoenix dactylifera* cultivars by PCO analysis

In MVSP, the results show that cultivars were classified into two groups in degree (0.292). The first group was divided into two clades in degree (0.454):1- Mabroom Al-Ula and Anbarah (0.780); 2- this clade was divided into two clades in degree (0.482): (a) Barni Al-Eis and Segaae were similar by (0.758) while Altaibat was similar by (0.605), (b) Barni Al-Madinah was similar by (0.500), Hilwah Al-Ula was similar by (0.586), Majdool and Safawi were similar by (0.789) and Shalabi was similar by (0.755) The second group was classified into two clades: 1- Loun was similar by (0.610), Beid and Rothanah were more similar by (0.963); 2- Ajwah was less similar by (0.335) (Figs. 10 and 11).

### Seeds

The characteristics of the seed were recorded in Table 6. The seed characters were different between cultivars. The range of seed length was from 3.4 cm (Mabroom Al-Ula) to 1.7 cm (Beid) while the range of leaflet width was from 0.83 cm (Beid and Barni Al-Eis) to 0.5 cm (Anbarah). In addition, the shapes of fruit were either ovoid, ovoid- oblong or linear- oblong. The tip of the seed was obtuse, apiculate or acute while the surface of the seed was either smooth or rough. The seed color was brown or dark brown. The measure was replicated in ten seeds in each palm.

**Table 6:** The seed characters of *Phoenix dactylifera* cultivars

Cultivars	Color	Seed Length LSD 0.07	Seed Width LSD 0.07	The ratio of Seed L/W	Shape	Tip of Seeds	Surface of Seed
Ajwah	Brown	2.18 ± 0.04**c**	0.65 ± 0.08 **cd**	3.35	Ovoid	Obtuse	Rough
Safawi	Brown	2.37 ± 0.05**e**	0.58 ± 0.04**b**	4.09	Ovoid	Apiculate	Smooth
Shalabi	Dark Brown	2.65 ± 0.08**f**	0.68 ± 0.04 **cd**	3.9	Ovoid-Oblong	Acute	Smooth
Rothanah	Brown	1.75 ± 0.05**a**	0.78 ± 0.04**f**	2.24	Ovoid-Oblong	Obtuse	Smooth
Barni Al-Madinah	Brown	2.9 ± 0.06 **g**	0.7 ± 0.06**e**	4.14	Ovoid	Acute	Rough
Segaae	Brown	2.3 ± 0.06**d**	0.62 ± 0.04**bc**	3.71	Ovoid- Oblong	Acute	Smooth
Majdool	Dark Brown	2.28 ± 0.04**d**	0.78 ± 0.07**f**	2.92	Ovoid- Oblong	Apiculate	Smooth
Loun	Brown	1.9 ± 0.06**b**	0.6 ± 0.06**bc**	3.17	Ovoid- Oblong	Obtuse	Smooth
Beid	Brown	1.7 ± 0.06**a**	0.83 ± 0.05**f**	2.05	Oblong-Ovoid	Obtuse	Rough
Barni Al-Eis	Brown	3.12 ± 0.08 **h**	0.83 ± 0.05**f**	3.76	Ovoid- Oblong	Acute	Rough
Anbarah	Dark Brown	2.92 ± 0.04 **g**	0.5 ± 0.06**a**	5.84	Linear -Oblong	Acute	Smooth
Hilwah Al-Ula	Brown	2.2 ± 0.06**c**	0.7 ± 0.06**e**	4.23	Ovoid- Oblong	Apiculate	Smooth
Altaibat	Brown	2.32 ± 0.04**de**	0.52 ± 0.04**a**	4.46	Ovoid- Oblong	Obtuse	Smooth
Mabroom Al-Ula	Dark Brown	3.4 ± 0.06**i**	0.6 ± 0.06**c**	5.67	Linear -Oblong	Apiculate	Smooth

In ANOVA test, there was a significant difference among them all two characters of seeds with a significance of (*p* < 0.001). LSD test was calculated to find the groups that have a significant difference among cultivars. The means of characters with the different letters were significantly different with *p* = 0.05 (Table 6). The fruit length was classified the cultivars into nine different groups (Rothanah, Beid), (Loun), (Ajwah, Hilwah Al-Ula), (Segaae, Majdool, Altaibat), (Safawi, Altaibat), (Shalabi), (Barni Al-Madinah, Anbarah), (Barni Al-Eis), (Mabroom) while the width of the seed was divided them into six groups (Anbarah, Altaibat), (Safawi, Segaae, Loun, Mabroom Al-Ula) (Altaibat, Segaae, Loun, Mabroom Al-Ula), (Ajwah, Shalabi), (Barni Al-Madinah, Hilwah Al-Ula) (Rothanah, Majdool, Beid, Barni Al-Eis) However, many cultivars were no significant differences between others in fruit width because one cultivar shared with others in several groups. Shalabi, Loun, Barni Al-Eis and Mabroom Al-Ula were distinguished in the length of their seeds.

**Fig. 12:**
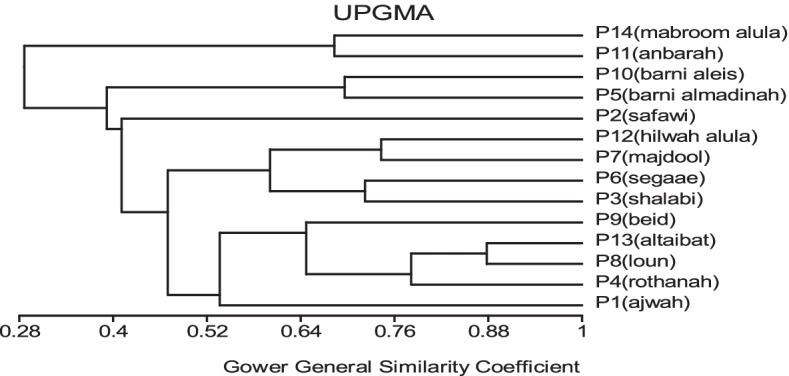
The degree of similarity and difference in the seed characteristics among *Phoenix dactylifera* cultivars by using cluster analysis

**Fig. 13:**
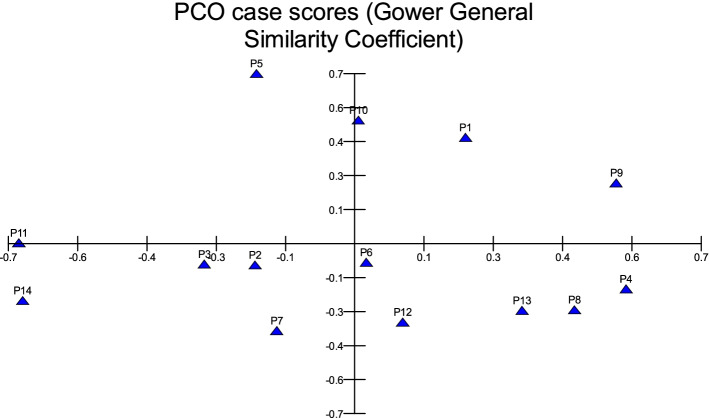
The degree of similarity and difference in the seed characteristics among *Phoenix dactylifera* cultivars by PCO analysis

In MVSP, the results show that cultivars were classified into two groups in degree (0.286). The first clade was Mabroom Al-Ula and Anbarah which were separated from the rest of the cultivars in degree (0.683). The second group was classified into two clades in degree (0.392): 1- Barni Al-Eis and Barni Al-Madinah were similar by (0.695); 2- this clade was divided into two clades: (a) Safawi was similar by (0.410), (b) this subgroup was divided into two clades in degree (0.470): (b1) Hilwah Al-Ula and Majdool were similar by (0.742), Segaae and Shalabi were similar by (0.722); (b2) Beid was similar by (0.647), Altaibat and Loun were more similar by (0.878) while Ajwah was similar by (0.537) (Figs. 12 and 13).

**Fig. 14:**
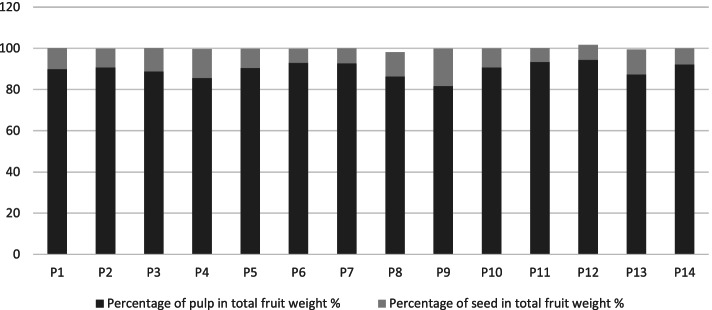
The variation between pulp of fruits and seeds in the total fruit weight in *Phoenix dactylifera* cultivars

Fruits and seeds’ weight in cultivars were recorded in Table 7. There was a difference among cultivars in the percentage of the pulp of fruits and the seeds in the total fruit weight (Fig. 14). The measure was replicated in ten fruits with their seeds in each palm.

**Table 7:** Fruits and seeds weight in *Phoenix dactylifera* cultivars

Cultivars	Fruit Weight (p + s)	Pulp Weight	Seed Weight	Percentage of pulp in total fruit weight %	Percentage of seed in total fruit weight %
Ajwah	9.75 ± 0.81	8.76 ± 0.86	0.99 ± 0.12	89.85	10.15
Safawi	9.38 ± 0.79	8.51 ± 0.7	0.86 ± 0.11	90.72	9.17
Shalabi	10.38 ± 0.92	9.22 ± 0.93	1.16 ± 0.06	88.82	11.18
Rothanah	6.67 ± 0.92	5.71 ± 0.8	0.94 ± 0.13	85.61	14.09
Barni Al-Madinah	9.85 ± 0.9	8.92 ± 0.82	0.91 ± 0.13	90.56	9.24
Segaae	9.64 ± 0.88	8.96 ± 0.71	0.66 ± 0.18	92.95	6.85
Majdool	13.07 ± 0.67	12.12 ± 0.77	0.94 ± 0.18	92.73	7.19
Loun	7.35 ± 0.64	6.35 ± 0.57	0.86 ± 0.16	86.39	11.70
Beid	5.83 ± 0.65	4.76 ± 0.57	1.06 ± 0.17	81.65	18.18
Barni Al-Eis	11.16 ± 0.74	10.12 ± 0.68	1.03 ± 0.34	90.68	9.23
Anbarah	11.42 ± 0.81	10.66 ± 0.81	0.77 ± 0.02	93.35	6.74
Hilwah Al-Ula	11.17 ± 0.71	10.55 ± 0.94	0.81 ± 0.14	94.45	7.25
Altaibat	4.74 ± 0.36	4.14 ± 0.42	0.57 ± 0.09	87.34	12.03
Mabroom Al-Ula	11.75 ± 0.88	10.82 ± 0.78	0.92 ± 0.13	92.09	7.83

### All morphological characteristics

**Fig. 15:**
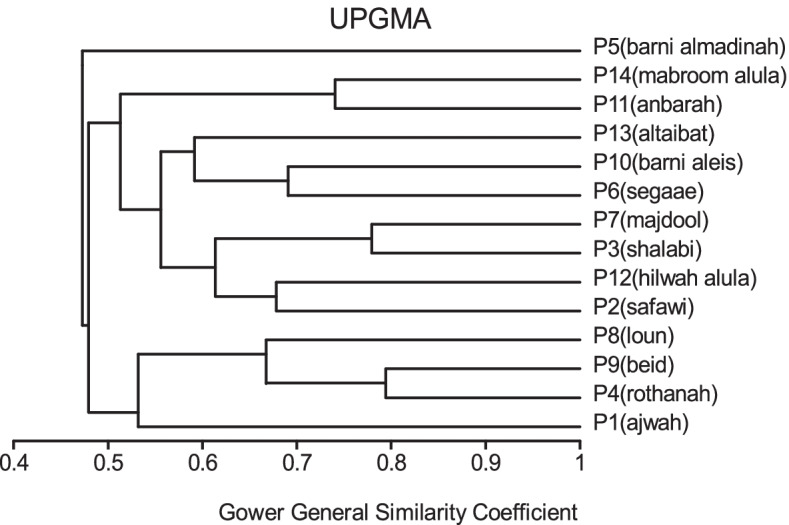
The degree of similarity and difference in all the morphological characteristics among *Phoenix dactylifera* cultivars by using cluster analysis

**Fig. 16:**
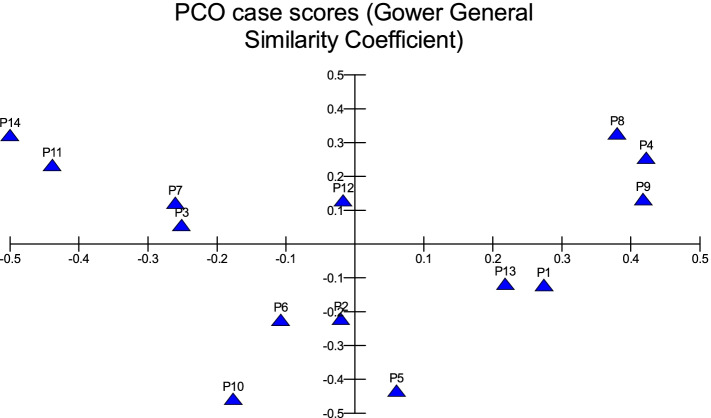
The degree of similarity and difference in all the morphological characteristics among *Phoenix dactylifera* cultivars by PCO analysis

All traits were added in cluster analysis and PCO analysis to compare the cultivars in total. Barni Al-Madinah was similar by (0.473). The rest of the cultivars were divided into two groups in degree (0.480). The first group was classified into two clades in degree (0.513): 1- Mabroom Al-Ula and Anbarah were similar by (0.740); 2- this subgroup was divided into two clades: (a) Barni Al-Eis and Segaae were similar by (0.691) while Altaibat was similar by (0.592), (b) Majdool and Shalabi were similar by (0.779) and Hilwah Al-Ula and Safawi were similar by (0.678). The second clade was divided into two clades:1- Beid and Rothanah were more similar (0.795) while Loun was similar by (0.667); 2- Ajwah was similar by (0.531) (Figs. 15 and 16).

## Discussion

Trunk features are useful to distinguish among cultivars. It is the most common feature. It is noticeable that no significant differences between Ajwah and Hilwah Al-Ula. However, it could be seen the differences among Barni Al-Eis, Segaae, Shalabi Altaibat, Anbarah, Loun and others. Circumference of the trunk divided the cultivars into groups and is in agreement with [11].

Frond features are useful to note the differences among cultivars. Altaibat and Barni Al-Madinah are different from the rest of the cultivars. However, it could be recognized the differences between Loun, Segaae, Beid, Barni Al-Eis, Ajwah, Safawi and others in frond characters. It was studied the morphological characteristics of Ajwah, Safawi and Anbarah and their result of frond length agreed with my study that they have differences from each other [6]. The length of frond shown the differences among cultivars and is in agreement with [3, 11]. The spined part length was a distinguished character and is in agreement with [8].

Leaflet features are helpful to distinguish among cultivars. Segaae and Shalabi are more related. On the other hand, it could be observed the differences between Mabroom Al-Ula, Barni Al-Madinah, Beid, Majdool and others in leaflet features. Ajwah was different from Anbarah and Safawi in the number of leaflets per frond [6]. However, Anbarah was different from Ajwah and Safawi in my study. The density of leaflets was grouped cultivars and is in agreement with [8, 11].

Spine characters are helpful to find the differences among cultivars. Hilwah Al-Ula and Safawi are closely related. However, it could be noted the differences between Majdool, Anbarah, Loun, Altaibat and others in spine characters. It was studied the morphological characteristics of Ajwah, Safawi and Anbarah, and their result of spine length were no significant differences among them [6], and is not in agreement with my result. Whereas the results of my study shown that Anbarah was different from Ajwah and Safawi in spines length. Also, they studied the number of spines per the frond, and their result shown that Safawi was different from Anbarah and Ajwah. In contrast, my result shown that Anbarah was different from Ajwah and Safawi. It indicated that were significant differences in length and number of spines in cultivars [11].

Fruit characters had a significant role to distinguish among cultivars. Beid and Rothanah are closely related. However, it could be distinguished the differences among Altaibat, Barni Al-Madinah, Hilwah Al-Ula, Shalabi, Loun, Ajwah and others in fruit characters. Fruit have diversity and differences in color, shape, length/width ratio, tip, base, and surface of the fruit, and this is in agreement with [4, 10]. It emphasized that the importance of fruit characters is to identify the cultivars [8].

Seed characters were helpful to differ among cultivars. Altaibat and Loun are more similar. However, it could be identified the differences between Safawi, Beid, Rothanah, Ajwah and others in seed characters. It reported that in addition to fruits, the features of the seed have an important role in comparing cultivars [8].

In all morphological characteristics, Barni Al-Madinah is the most distinguished cultivar from the rest of the cultivars. Mabroom Al-Ula and Anbarah are more related. Barni Al-Eis and Segaae are similar. Majdool and Shalabi are related. Hilwah Al-Ula and Safawi are similar. Beid and Rothanah are similar. In contrast, the least related are Altaibat, Loun, and Ajwah.

Thus, the main distinguishing characters identify each cultivar: Ajwah in fruit and seed characters; Rothanah in seed characters; Beid in frond and leaflet characters; Loun in trunk, frond, spines and fruit characters; Safawi in frond and seed characters; Hilwah Al-Ula in fruit characters; Shalabi in trunk and fruit characters; Majdool in leaflet and spine characters; Segaae in trunk and frond characters; Barni Al-Eis in trunk and frond characters; Altaibat in the trunk, spines and fruit characters; Anbarah in trunk and spines characters; Mabroom Al-Ula in leaflet characters; Barni Al-Madinah in leaflet and fruit characters.

## Conclusions

**Fig. 17:**
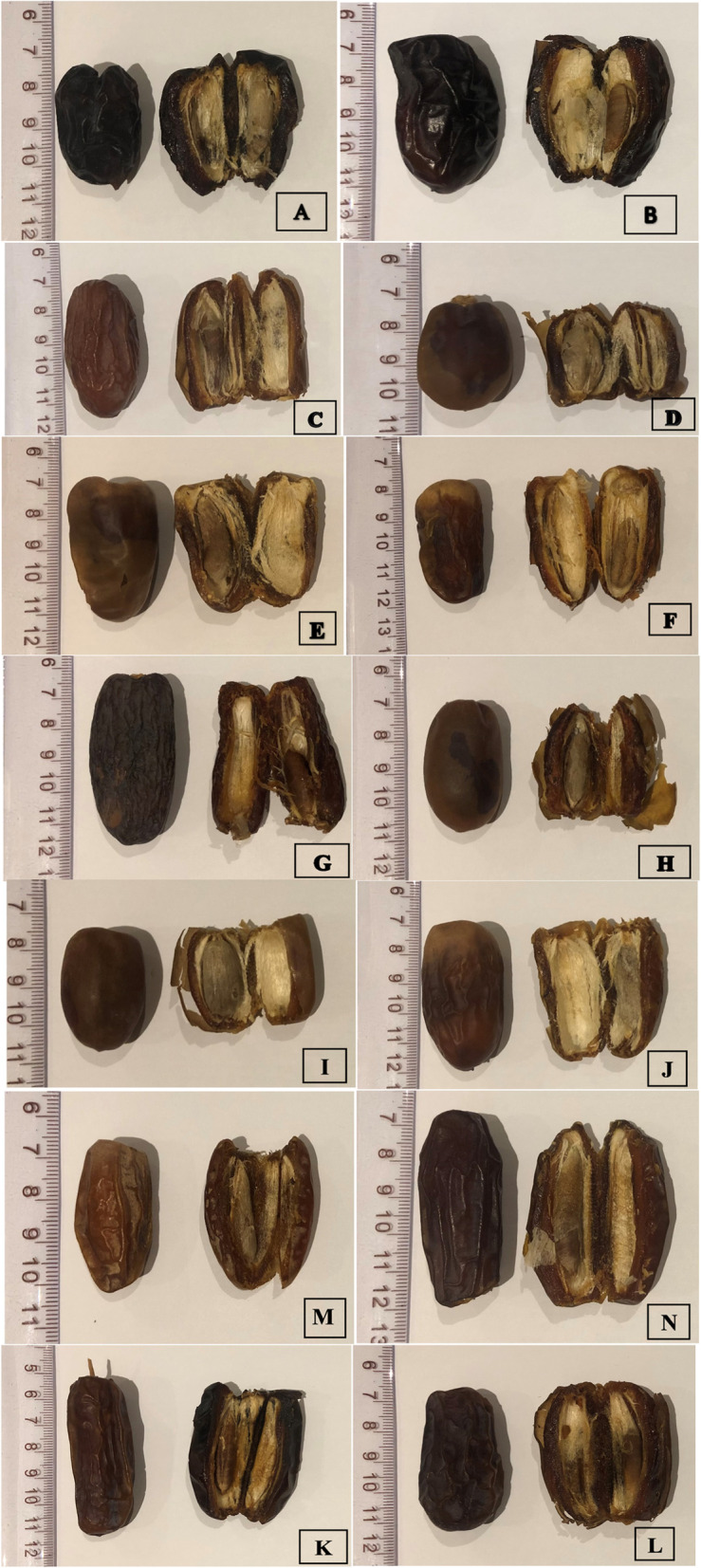
The fruit of *Phoenix dactylifera* cultivars (**A **Ajwah, **B **Safawi, **C **Shalabi, **D **Rothanah, **E **Barni Al-Madinah, **F **Segaae, **G **Majdool, **H **Loun, **I **Beid, **J **Barni Al-Eis, **K **Anbarah, **L **Hilwah Al-Ula, **M **Altaibat, **N **Mabroom Al-Ula)

**Fig. 18:**
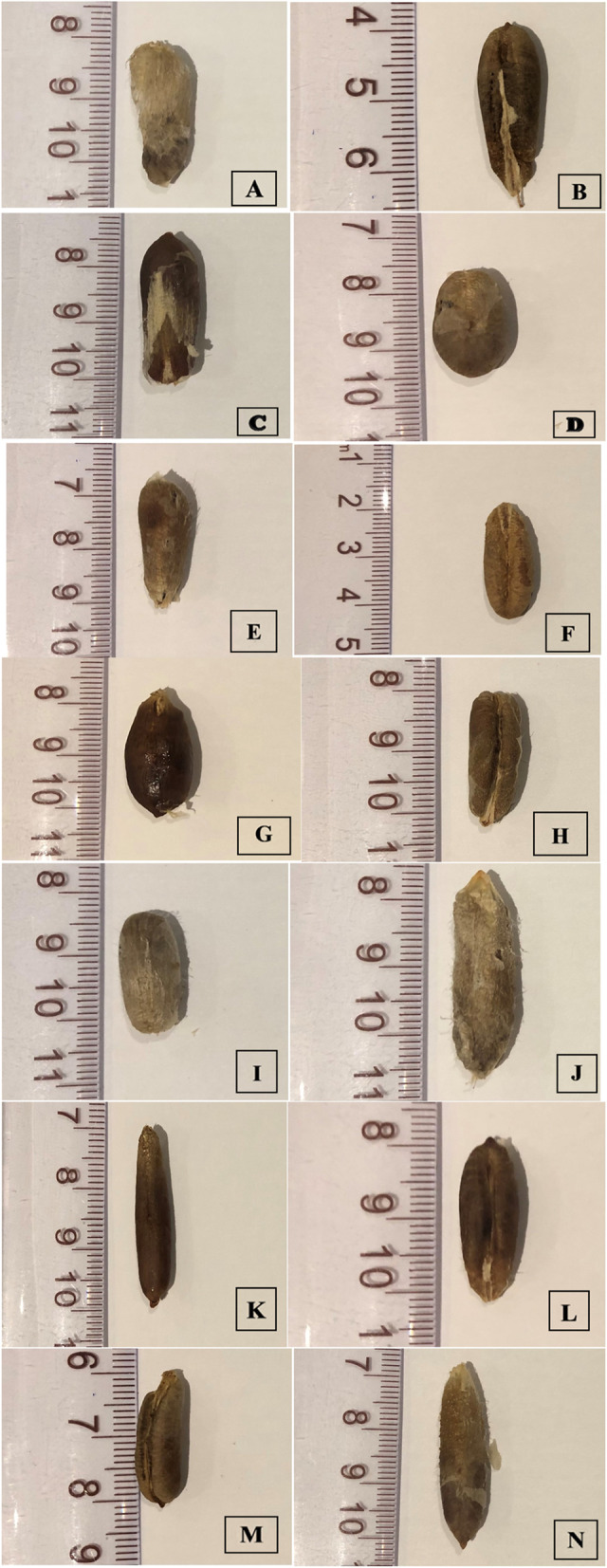
The seed of *Phoenix dactylifera* cultivars (**A **Ajwah, **B **Safawi, **C **Shalabi, **D **Rothanah, **E **Barni Al-Madinah, **F **Segaae, **G **Majdool, **H **Loun, **I **Beid, **J **Barni Al-Eis, **K **Anbarah, **L **Hilwah Al-Ula, **M **Altaibat, **N **Mabroom Al-Ula)

Based on the distances of similarity and differences among cultivars, the most distinguishing characteristics that can be useful to differentiate between cultivars are fruit and seed characters (Figs. 17 and 18), and the least features are trunk characters. However, fronds, leaflets, and spines characters create the differences between cultivars depending on the distances of similarity and differences. As a result, each morphological character may have a significant role to identify a certain cultivar.

## Methods

### Field work

**Fig. 19:**
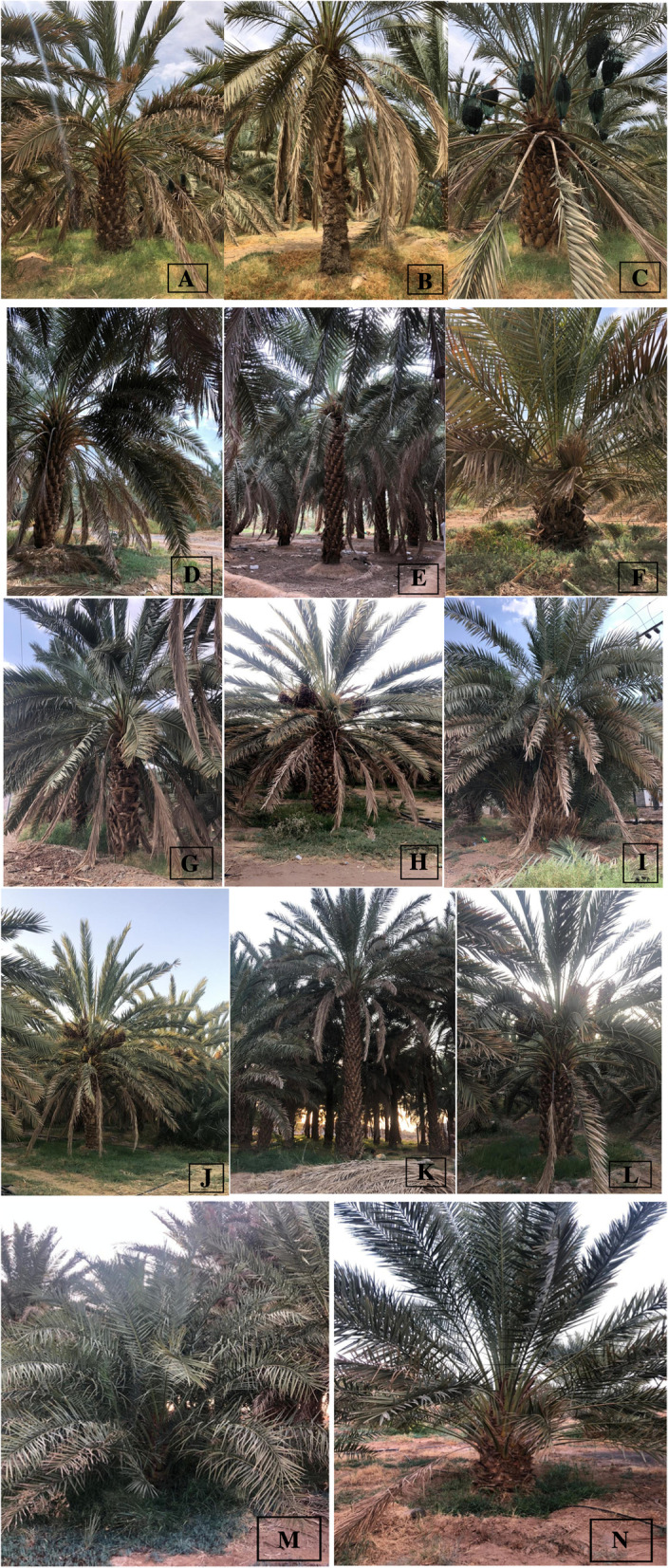
*Phoenix dactylifera* cultivars in Al-Madinah (**A **Ajwah, **B **Safawi, **C **Shalabi, **D **Rothanah, **E **Barni Al-Madinah, **F **Segaae, **G **Majdool, **H **Loun, **I **Beid, **J **Barni Al-Eis, **K **Anbarah, **L **Hilwah Al-Ula, **M **Altaibat, **N **Mabroom Al-Ula)

Plant specimens were collected in September 2021 from different areas in Al-Madinah Al-Munawarah region (Al-Madinah city, Al-Eis, Khaiber and Al-Ula) (Fig. 19). The collection of plant permission was granted from the respective authority. The date of collection, location, altitude, latitude and longitude, collection number and type of soil were noted in Table 8.

**Table 8:** The collection information of date palm cultivars in Al-Madinah region

Collection No.	Cultivars	Date	Location	Coordinates	Altitude	Soil Type
P1	Ajwah	Sep.22	Al-Madinah	24.600304-39.433634	543 m	Clay-sandy
P2	Safawi	Sep.22	Al-Madinah	24.593197-39.491828	669 m	Clay-sandy
P3	Shalabi	Sep.22	Al-Madinah	24.600599-39.433675	544 m	Clay-sandy
P4	Rothanah	Sep.22	Al-Madinah	24.600630- 39.434389	543 m	Clay-sandy
P5	Barni Al-Madinah	Sep.22	Al-Madinah	24.599862- 39.434486	543 m	Clay-sandy
P6	Segaae	Sep.22	Al-Madinah	24.599127- 39.435675	544 m	Clay-sandy
P7	Majdool	Sep.22	Al-Madinah	24.599383-39.435422	544 m	Clay-sandy
P8	Loun	Sep.22	Al-Madinah	24.600262- 39.435240	543 m	Clay-sandy
P9	Beid	Sep.22	Al-Madinah	24.599468-39.433910	544 m	Clay-sandy
P10	Barni Al-Eis	Sep.23	Al-Eis	25.068037-38.110739	618 m	Sandy
P11	Anbarah	Sep.24	Khaiber	25.914428-39.385451	778 m	Sandy
P12	Hilwah Al-Ula	Sep.24	Al-Ula	26.457493-38.064144	585 m	Sandy
P13	Altaibat	Sep.25	Khaiber	25.914502-39.385447	777 m	Sandy
P14	Mabroom Al-Ula	Sep.25	Al-Ula	26.457493-38.064144	584 m	Sandy

### Herbarium work

Specimens were pressed as quickly as possible after collection by folding them in sheets of newspaper and placing them in the press. Fruits were kept in Alcohol 70% for preservation. Plant specimens were kept in the King Abdulaziz herbarium (KAUH), and voucher specimens (Alaida, 1-14) were deposited in KAUH. The samples were identified according to wildflowers of Saudi Arabia [12] and by experts.

### Morphological work

The distinguished characters were recorded, such as trunk (diameter of trunk and trunk circumference), leaves (color, length, width, length of pinnated part, length of spined part, percentage of pinnated and spined parts of total leaf length, length and width of pinnae, length to width ratio of pinnae, number of pinnae per leaf, length of spines and number of spines per leaf), fruits(color of fresh and dry fruits, length, width, shape, length to width ratio, tip of the fruit, weight of pulp, base of the fruit and surface), and seeds (shape, length, width, color, weight, length to width ratio, surface and tip of seeds). The target of focusing on these characteristics was the ability to note between individuals palms easily and clearly, and the morphological characteristics were determined according to the methods used by [13, 14].

### Statistical analyses

All the data obtained from morphology was transferred to numerical characters and used in the multivariate statistical package (MVSP) to study the similarity between the cultivars and give phenetic clusters. All the data was transferred to numerical values in a matrix table to analyze and draw scatterplots and dendrograms (Table 9). In addition, one- way ANOVA test and multi-comparative test were used to find the significant differences among cultivars in *p* = 0.05.

**Table 9:** The data matrix of all morphological characteristics of *Phoenix dactylifera* cultivars

Character	Character States
Diameter of Trunk	Numerical values
Trunk Circumference	Numerical values
Length of Frond	Numerical values
Width of Frond	Numerical values
Pinnated Part Length	Numerical values
Spined Part Length	Numerical values
Color of Leaflets	Multi-State (0 = Light Green, 1 = Dark Green, 2 = Ashy Green)
Length of leaflet	Numerical values
Width of leaflet	Numerical values
Number of Leaflets per Frond	Numerical values
Length of Spines	Numerical values
Number of Spines per Frond	Numerical values
Color of Fresh Fruit	Binary (0 = Yellow, 1 = Red)
Color of Dry Fruit	Multi-State (0 = Brown, 1 = Light Brown, 2 = Reddish Brown, 3 = Black)
Fruit Length	Numerical values
Fruit Width	Numerical values
Fruit Shape	Multi-State (0 = Globose, 1 = Ovoid, 2 = Oblong, 3 = Linear-Oblong)
Tip of the Fruit	Multi-State (0 = Rounded, 1 = Cordate, 2 = Shallowly cordate, 3 = Truncate)
The base of the Fruit	Binary (0 = Rounded, 1 = Obtuse)
Surface of Fruit	Binary (0 = Smooth, 1 = Rugose)
Color of Seed	Binary (0 = Brown, 1 = Dark Brown)
Seed Length	Numerical values
Seed Width	Numerical values
Seed Shape	Multi-State (0 = Globose, 1 = Ovoid, 2 = Ovoid-Oblong, 3 = Linear -Oblong)
Tip of Seeds	Multi-State (0 = Obtuse, 1 = Acute, 2 = Apiculate)
Surface of Seed	Binary (0 = Smooth, 1 = Rugose)

## Data Availability

All data analyzed or generated in this study are included in this published article.
